# Entropy and Stability: Reduced Hamiltonian Formalism of Non-Barotropic Flows and Instability Constraints

**DOI:** 10.3390/e27080779

**Published:** 2025-07-23

**Authors:** Asher Yahalom

**Affiliations:** 1 Department of Electrical & Electronic Engineering, Faculty of Engineering, Ariel University, Ariel 40700, Israel; asya@ariel.ac.il; Tel.: +972-54-7740294; 2 Center for Astrophysics, Geophysics, and Space Sciences (AGASS), Ariel University, Ariel 40700, Israel; 3 Free Electron Lasers (FEL) User Center, Ariel University, Ariel 40700, Israel

**Keywords:** non-barotropic flows, variational analysis, topological conservation laws

## Abstract

A reduced representation of a dynamical system helps us to understand what the true degrees of freedom of that system are and thus what the possible instabilities are. Here we extend previous work on barotropic flows to the more general non-barotropic flow case and study the implications for variational analysis and conserved quantities of topological significance such as circulation and helicity. In particular we introduce a four-function Eulerian variational principle of non-barotropic flows, which has not been described before. Also new conserved quantities of non-barotropic flows related to the topological velocity field, topological circulation and topological helicity, including a local version of topological helicity, are introduced. The variational formalism given in terms of a Lagrangian density allows us to introduce canonical momenta and hence a Hamiltonian formalism.

## 1. Introduction

A comprehensive introduction to the subject of variational principles of fluid dynamics is given in [1] and will not be reproduced here; the reader who is interested in a broader introduction is referred to the original paper. We shall mention, however, that parts of this paper, especially Section 5, follow closely the work of Salmon ([2], Section 3). Also we take notice of the remarkable papers by Holm, Marsden, and Ratiu [3] and Morrison [4], who address the issue from an abstract point of view.

In this paper we extend what is known on variational barotropic fluid dynamics to non-barotropic fluid dynamics; this also has an impact on the form of conserved quantities such as circulation and helicity, which have a well-known topological meaning in terms of the knottiness of vortex lines [5].

Non-barotropic flows are distinguished from barotropic flows by their more realistic equation of state. The internal energy of these flows, and therefore the pressure, depend on both the density and specific entropy in contrast to the (over)simplified equation of state of a barotropic flow, which is a function of density alone. This formulation allows us to examine how entropy and temperature influence flow dynamics and paves the way for future developments towards the variational analysis of non-ideal flows in which heat losses play an important role.

We begin by introducing the basic equations. This is followed by a discussion of the Lagrangian variational principle for fluid dynamics. Then we discuss the Eulerian variational principle and its simplification, including its stationary form. This is followed by the definition of canonical momenta and derivation of the Hamiltonian of the system. The next step is the analysis of conservation laws within the non-barotropic framework. Then we demonstrate that the non-barotropic variational problem can be formulated in terms of only four functions. Finally, we give an analytic solution of a family of flows, which also includes self-gravitating tori.

## 2. Fundamental Equations of Non-Stationary Non-Barotropic Fluid Dynamics

Non-barotropic Eulerian flows are described using five dependent variables, the density ρ, velocity $\vec{v}$, and specific entropy *s*. These variables satisfy the continuity and Euler equations and ideal flow requirement, which is the lack of heat losses in every fluid element:

(1) $$\frac{\partial \rho }{\partial t}+\vec{\nabla }\cdot \left(\rho \vec{v}\right)=0$$

(2) $$\frac{d\vec{v}}{dt}\equiv \frac{\partial \vec{v}}{\partial t}+\left(\vec{v}\cdot \vec{\nabla }\right)\vec{v}=-\frac{\vec{\nabla }p\left(\rho ,s\right)}{\rho }-\vec{\nabla }\Psi$$

(3) $$\frac{ds}{dt}\equiv \frac{\partial s}{\partial t}+\left(\vec{v}\cdot \vec{\nabla }\right)s=0$$

The pressure function p(ρ,s) is dependent on the density ρ and specific entropy *s*, Ψ is some specific force potential (which can be gravitational or electromagnetic), $\frac{\partial }{\partial t}$ is a partial time derivative, and $\vec{\nabla }$ is the nabla operator of vector analysis. The derivative $\frac{d}{dt}$ is the material time derivative. Equation (1) tells us that the mass of each fluid element is conserved, Equation (2) is just Newton’s second law for continuous matter, while Equation (3) is a mathematical expression for the fact that in an ideal flow, heat is not conducted nor created but only convected.

### Summary

In this section we introduce the basic notations and equations that are used in the mathematical description of non-barotropic fluid dynamics.

## 3. Thermodynamics and Vortex Dynamics

Applying the rotor operator to Equation (2) will lead to

(4) $$\frac{\partial \vec{\omega }}{\partial t}=\vec{\nabla }\times \left(\vec{v}\times \vec{\omega }\right)-\vec{\nabla }\times \left(\frac{\vec{\nabla }p}{\rho }\right)$$

in which

(5) $$\vec{\omega }=\vec{\nabla }\times \vec{v}$$

is the vorticity. Let us now look at the thermodynamics of the fluid. The fluid consists of “fluid elements” [6,7] which are conceptualized as “point particles” with certain properties. Each “fluid element” has an infinitesimal mass $dM_{\vec{\alpha }}$, a position vector $\vec{x}\left(\vec{\alpha },t\right)$, and a velocity $\vec{v}\left(\vec{\alpha },t\right)\equiv \frac{d\vec{x}\left(\vec{\alpha },t\right)}{dt}$. Unlike true point particles, these fluid elements also possess an infinitesimal volume $dV_{\vec{\alpha }}$, an infinitesimal amount of entropy $dS_{\vec{\alpha }}$, and an infinitesimal internal energy $dE_{in\;\vec{\alpha }}$. It is common practice to define densities for the Lagrangian and mass of each fluid element in this context:

(6) $$\rho_{\vec{\alpha }}\equiv \frac{dM_{\vec{\alpha }}}{dV_{\vec{\alpha }}}.$$

The density is dependent on $\vec{x}$, where the “fluid element” labeled $\vec{\alpha }$ is in time *t*:

(7) $$\rho \left(\vec{x},t\right)\equiv \rho \left(\vec{x}\left(\vec{\alpha },t\right),t\right)\equiv \rho_{\vec{\alpha }}\left(t\right)$$

It is also common practice to define the specific internal energy $\varepsilon_{\vec{\alpha }}$:

(8) $$\varepsilon_{\vec{\alpha }}\equiv \frac{dE_{in\;\vec{\alpha }}}{dM_{\vec{\alpha }}}\;\Rightarrow \;\rho_{\vec{\alpha }}\varepsilon_{\vec{\alpha }}=\frac{dM_{\vec{\alpha }}}{dV_{\vec{\alpha }}}\frac{dE_{in\;\vec{\alpha }}}{dM_{\vec{\alpha }}}=\frac{dE_{in\;\vec{\alpha }}}{dV_{\vec{\alpha }}}$$

which will be important later in the current paper. In an ideal case the “fluid element” does not exchange mass nor heat with other fluid elements; thus

(9) $$\Delta dM_{\vec{\alpha }}=\Delta dS_{\vec{\alpha }}=0.$$

in which we use the Δ symbol to denote change. According to thermodynamics a modification of the “fluid element” internal energy satisfies

(10) $$\Delta dE_{in\;\vec{\alpha }}=T_{\vec{\alpha }}\Delta dS_{\vec{\alpha }}-p_{\vec{\alpha }}\Delta dV_{\vec{\alpha }},$$

the first term on the right-hand side of the above equation describes the heat gained by the “fluid element”, while the second term on the right-hand side is the work done by the “fluid element” on surrounding elements. In the above $T_{\vec{\alpha }}$ denotes the temperature of the “fluid element” and $p_{\vec{\alpha }}$ is its pressure. As the mass of the fluid element is constant, we may divide Equation (10) by $dM_{\vec{\alpha }}$ to derive the variation in the specific energy:

(11) $$\begin{array}{ccc} \Delta \varepsilon_{\vec{\alpha }} & = & \Delta \frac{dE_{in\;\vec{\alpha }}}{dM_{\vec{\alpha }}}=T_{\vec{\alpha }}\Delta \frac{dS_{\vec{\alpha }}}{dM_{\vec{\alpha }}}-p_{\vec{\alpha }}\Delta \frac{dV_{\vec{\alpha }}}{dM_{\vec{\alpha }}}\\ & = & T_{\vec{\alpha }}\Delta s_{\vec{\alpha }}-p_{\vec{\alpha }}\Delta \frac{1}{\rho_{\vec{\alpha }}}=T_{\vec{\alpha }}\Delta s_{\vec{\alpha }}+\frac{p_{\vec{\alpha }}}{\rho_{\vec{\alpha }}^{2}}\Delta \rho_{\vec{\alpha }}.\;s_{\vec{\alpha }}\equiv \frac{dS_{\vec{\alpha }}}{dM_{\vec{\alpha }}} \end{array}$$

$s_{\vec{\alpha }}$ is the specific entropy. Thus,

(12) $$\frac{\partial \varepsilon }{\partial s}=T,\;\frac{\partial \varepsilon }{\partial \rho }=\frac{p}{\rho^{2}}.$$

The enthalpy is defined as follows:

(13) $$dW_{\vec{\alpha }}=dE_{in\;\vec{\alpha }}+p_{\vec{\alpha }}dV_{\vec{\alpha }}.$$

and thus the specific enthalpy can be calculated as follows:

(14) $$w_{\vec{\alpha }}=\frac{dW_{\vec{\alpha }}}{dM_{\vec{\alpha }}}=\frac{dE_{in\;\vec{\alpha }}}{dM_{\vec{\alpha }}}+p_{\vec{\alpha }}\frac{dV_{\vec{\alpha }}}{dM_{\vec{\alpha }}}=\varepsilon_{\vec{\alpha }}+\frac{p_{\vec{\alpha }}}{\rho_{\vec{\alpha }}}.$$

Thus using Equation (12) we obtain:

(15) $$w=\varepsilon +\frac{p}{\rho }=\varepsilon +\rho \frac{\partial \varepsilon }{\partial \rho }=\frac{\partial (\rho \varepsilon )}{\partial \rho }.$$

Moreover,

(16) $$\frac{\partial w}{\partial \rho }=\frac{\partial (\varepsilon +\frac{p}{\rho })}{\partial \rho }=-\frac{p}{\rho^{2}}+\frac{1}{\rho }\frac{\partial p}{\partial \rho }+\frac{\partial \varepsilon }{\partial \rho }=-\frac{p}{\rho^{2}}+\frac{1}{\rho }\frac{\partial p}{\partial \rho }+\frac{p}{\rho^{2}}=\frac{1}{\rho }\frac{\partial p}{\partial \rho }.$$

Now we can look again at the gradient of the pressure appearing in Equation (4):

(17) $$\frac{\vec{\nabla }p}{\rho }=\vec{\nabla }\left(\frac{p}{\rho }\right)+\frac{p}{\rho^{2}}\vec{\nabla }\rho =\vec{\nabla }\left(w-\varepsilon \right)+\frac{p}{\rho^{2}}\vec{\nabla }\rho$$

in which we have used Equation (15) in the above derivation. Looking again at Equation (11), and considering a change of the fluid element location, leads to the following identity for the specific internal energy gradient:

(18) $$\vec{\nabla }\varepsilon =T\vec{\nabla }s+\frac{p}{\rho^{2}}\vec{\nabla }\rho .$$

Combining Equation (17) with Equation (18) leads to the equation:

(19) $$\frac{\vec{\nabla }p}{\rho }=\vec{\nabla }w-T\vec{\nabla }s$$

We can now plug the above equation into Equation (4) to obtain:

(20) $$\frac{\partial \vec{\omega }}{\partial t}=\vec{\nabla }\times \left(\vec{v}\times \vec{\omega }\right)+\vec{\nabla }T\times \vec{\nabla }s.$$

Equation (4) is a mathematical statement about the vorticity lines being “frozen” in the flow if the temperature or specific entropy are uniform or their gradients are parallel. Frozen vorticity lines (or not) are obviously connected to the conservation of circulation and helicity and thus signify the profound connection in flows between thermodynamics and vortex dynamics.

### Summary

In this section we elaborate on the thermodynamics of a fluid element, introducing the relevant concepts that will serve us later in this paper; we also demonstrate the connection between vortex dynamics and thermodynamics.

## 4. The Lagrangian Variational Approach

The Lagrangian and action for every “fluid element” are the same as those of a point particle, with one difference: its internal energy. The variational formalism of point particles is described in Appendix A. Thus according to Equation (A6) we obtain the expression:

(21) $$\begin{array}{ccc} dA_{\vec{\alpha }} & = & \int_{t1}^{t2}dL_{\vec{\alpha }}dt,\\ dL_{\vec{\alpha }} & \equiv & \frac{1}{2}dM_{\vec{\alpha }}\;v{\left(\vec{\alpha },t\right)}^{2}-dM_{\vec{\alpha }}\Psi_{\vec{\alpha }}-dE_{in\;\vec{\alpha }}. \end{array}$$

in which we replace the discrete index *j* with the continuous index $\vec{\alpha }$. Of course, all quantities are calculated for a unique $\vec{\alpha }$. The fluid’s Lagrangian and action are summed (or integrated) over all $\vec{\alpha }$’s:

(22) $$\begin{array}{ccc} L & = & \int_{\vec{\alpha }}dL_{\vec{\alpha }}\\ A & = & \int_{\vec{\alpha }}dA_{\vec{\alpha }}=\int_{t1}^{t2}\int_{\vec{\alpha }}dL_{\vec{\alpha }}dt=\int_{t1}^{t2}Ldt. \end{array}$$

It follows that the Lagrangian density is:

(23) $$\begin{array}{ccc} L_{\vec{\alpha }} & = & \frac{dL_{\vec{\alpha }}}{dV_{\vec{\alpha }}}\\ L_{\vec{\alpha }} & \equiv & \frac{1}{2}\rho_{\vec{\alpha }}v{\left(\vec{\alpha },t\right)}^{2}-\rho_{\vec{\alpha }}\varepsilon_{\vec{\alpha }}-\rho_{\vec{\alpha }}\Psi_{\vec{\alpha }}. \end{array}$$

The Lagrangian is thus a spatial integral:

(24) $$L=\int_{\vec{\alpha }}dL_{\vec{\alpha }}=\int_{\vec{\alpha }}L_{\vec{\alpha }}dV_{\vec{\alpha }}=\int L\left(\vec{x},t\right)d^{3}x$$

We shall now vary the action. The only term different from the classical case is the internal energy term, whose variation is given in Equation (10). For an ideal fluid, heat is not generated nor is heat conducted or radiated, and thus heat can only be convected by the “fluid elements”. It follows that $\Delta dS_{\vec{\alpha }}=0$ and thus:

(25) $$\Delta dE_{in\;\vec{\alpha }}=-p_{\vec{\alpha }}\Delta dV_{\vec{\alpha }}.$$

We now vary the volume element:

(26) $$dV_{\vec{\alpha },t}=d^{3}x\left(\vec{\alpha },t\right)$$

With the help of the Jacobian, we relate this to the volume element at t=0 (see also [8], Equations (2.8) and (2.14)):

(27) $$d^{3}x\left(\vec{\alpha },t\right)=Jd^{3}x\left(\vec{\alpha },0\right),\;J\equiv {\vec{\nabla }}_{0}x_{1}\cdot \left({\vec{\nabla }}_{0}x_{2}\times {\vec{\nabla }}_{0}x_{3}\right)$$

${\vec{\nabla }}_{0}\equiv \left(\frac{\partial }{\partial x{\left(\vec{\alpha },0\right)}_{1}},\frac{\partial }{\partial x{\left(\vec{\alpha },0\right)}_{2}},\frac{\partial }{\partial x{\left(\vec{\alpha },0\right)}_{3}}\right)$. It follows from Equations (A9) and (A12) that the variation in the volume of each “fluid element” is:

(28) $$\Delta dV_{\vec{\alpha },t}=\vec{\nabla }\cdot \vec{\xi }\;dV_{\vec{\alpha },t}.$$

And thus the variation in the internal energy follows:

(29) $$\Delta dE_{in\;\vec{\alpha }}=-p\vec{\nabla }\cdot \vec{\xi }\;dV_{\vec{\alpha },t}.$$

The internal energy is the main new aspect compared to the single-particle or system of particles scenarios described in the Appendix. Therefore, the rest of the variation analysis proceeds in a straightforward manner. Varying Equation (21) we derive:

(30) $$\begin{array}{ccc} \Delta dA_{\vec{\alpha }} & = & \int_{t1}^{t2}\Delta dL_{\vec{\alpha }}dt,\\ \Delta dL_{\vec{\alpha }} & = & dM_{\vec{\alpha }}\;\left(\vec{v}\left(\vec{\alpha },t\right)\cdot \Delta \vec{v}\left(\vec{\alpha },t\right)-\Delta \Psi_{\vec{\alpha }}\right)-\Delta dE_{in\;\vec{\alpha }}. \end{array}$$

Notice that:

(31) $$\Delta \vec{v}\left(\vec{\alpha },t\right)=\Delta \frac{d\vec{x}\left(\vec{\alpha },t\right)}{dt}=\frac{d\Delta \vec{x}\left(\vec{\alpha },t\right)}{dt}=\frac{d\vec{\xi }\left(\vec{\alpha },t\right)}{dt}.$$

After the steps that lead to Equation (A2) we obtain:

(32) $$\Delta dL_{\vec{\alpha }}=\frac{d(dM_{\vec{\alpha }}{\vec{v}}_{\vec{\alpha }}\cdot {\vec{\xi }}_{\vec{\alpha }})}{dt}-dM_{\vec{\alpha }}\left(\frac{d{\vec{v}}_{\vec{\alpha }}}{dt}+\vec{\nabla }\Psi_{\vec{\alpha }}\right)\cdot {\vec{\xi }}_{\vec{\alpha }}+p\vec{\nabla }\cdot {\vec{\xi }}_{\vec{\alpha }}\;dV_{\vec{\alpha },t}.$$

The variation in a single element’s action is thus:

(33) $$\begin{array}{ccc} \Delta dA_{\vec{\alpha }} & = & \int_{t1}^{t2}\Delta dL_{\vec{\alpha }}dt={\left.dM_{\vec{\alpha }}\vec{v}\left(\vec{\alpha },t\right)\cdot {\vec{\xi }}_{\vec{\alpha }}\right|}_{t1}^{t2}\\ & - & \int_{t1}^{t2}\left(dM_{\vec{\alpha }}\left(\frac{d{\vec{v}}_{\vec{\alpha }}}{dt}+\vec{\nabla }\Psi_{\vec{\alpha }}\right)\cdot {\vec{\xi }}_{\vec{\alpha }}-p\vec{\nabla }\cdot {\vec{\xi }}_{\vec{\alpha }}\;dV_{\vec{\alpha },t}\right)dt. \end{array}$$

From the above we easily derive the total action variation:

(34) $$\begin{array}{ccc} \Delta A & = & \Delta \int_{\vec{\alpha }}dA_{\vec{\alpha }}={\left.\int_{\vec{\alpha }}dM_{\vec{\alpha }}\vec{v}\left(\vec{\alpha },t\right)\cdot {\vec{\xi }}_{\vec{\alpha }}\right|}_{t1}^{t2}\\ & - & \int_{t1}^{t2}\int_{\vec{\alpha }}\left(dM_{\vec{\alpha }}\left(\frac{d{\vec{v}}_{\vec{\alpha }}}{dt}+\vec{\nabla }\Psi_{\vec{\alpha }}\right)\cdot {\vec{\xi }}_{\vec{\alpha }}-p\vec{\nabla }\cdot {\vec{\xi }}_{\vec{\alpha }}\;dV_{\vec{\alpha }}\right)dt. \end{array}$$

Now according to Equation (6) we may write:

(35) $$dM_{\vec{\alpha }}=\rho_{\vec{\alpha }}\;dV_{\vec{\alpha }},$$

and thus convert the $\vec{\alpha }$ integral into a volume integral:

(36) $$\Delta A={\left.\int \rho \vec{v}\cdot \vec{\xi }dV\right|}_{t1}^{t2}-\int_{t1}^{t2}\int \left(\rho \left(\frac{d\vec{v}}{dt}+\vec{\nabla }\Psi \right)\cdot \vec{\xi }-p\vec{\nabla }\cdot \vec{\xi }\right)dVdt.$$

According to the identity:

(37) $$p\vec{\nabla }\cdot \vec{\xi }=\vec{\nabla }\cdot \left(p\vec{\xi }\right)-\vec{\xi }\cdot \vec{\nabla }p,$$

with the help of Gauss’s theorem we write:

(38) $$\begin{array}{ccc} \Delta A & = & {\left.\int \rho \vec{v}\cdot \vec{\xi }dV\right|}_{t1}^{t2}\\ & - & \int_{t1}^{t2}\left[\int \left(\rho \left(\frac{d\vec{v}}{dt}+\vec{\nabla }\Psi \right)+\vec{\nabla }p\right)\cdot \vec{\xi }dV-\oint p\vec{\xi }\cdot d\vec{\Sigma }\right]dt. \end{array}$$

The action variation will vanish if $\vec{\xi }\left(t1\right)=\vec{\xi }\left(t2\right)=0$, and the otherwise arbitrary $\vec{\xi }$ vanishes on the surface surrounding the fluid only if the Euler equation is satisfied:

(39) $$\frac{d\vec{v}}{dt}=-\frac{\vec{\nabla }p}{\rho }-\vec{\nabla }\Psi ,\;\frac{d}{dt}=\frac{\partial }{\partial t}+\vec{v}\cdot \vec{\nabla }$$

The equation, apart from the pressure terms, resembles that of a point particle. In experimental fluid dynamics, it is often more practical to describe a fluid using quantities at specific locations rather than those associated with unseen infinitesimal “fluid elements”. This approach leads to the Eulerian description of fluid dynamics, which focuses on flow fields rather than the velocities of individual “fluid elements”; this will be investigated in Section 5.

### Summary

In this section we introduce an action principle given in the Lagrangian picture of fluid dynamics. It is shown how the equations of motion can be derived from this action principle using the calculus of variations.

## 5. The Eulerian Variational Principle

The Eulerian approach is a radically different way to study flows. Instead of fluid elements we look at fluid fields, which may be scalar, such as the density of mass $\rho (\vec{x},t)$ and the entropy per unit mass (specific entropy) $s(\vec{x},t)$, or vector, such as the velocity field $\vec{v}\left(\vec{x},t\right)$. To achieve this we will need to add auxiliary functions for maintaining information that is lost in the transformation from the Lagrangian to the Eulerian picture, such as the conservation of the particles’ mass and identity. Here we follow in the footsteps of Clebsch [9,10], Davydov [11], and Seliger & Whitham [12]. We write the action:

(40) $$\begin{array}{ccc} A & \equiv & \int Ld^{3}xdt\\ L & \equiv & L_{1}+L_{2}\\ L_{1} & \equiv & \rho \left(\frac{1}{2}{\vec{v}}^{2}-\varepsilon \left(\rho ,s\right)-\Psi \right),\;L_{2}\equiv \nu \left[\frac{\partial \rho }{\partial t}+\vec{\nabla }\cdot \left(\rho \vec{v}\right)\right]-\rho \alpha \frac{d\beta }{dt}-\rho \sigma \frac{ds}{dt}. \end{array}$$

In the above $L_{1}$ is (23). $L_{2}$ is composed of a set of constraints that are enforced by the Lagrange multipliers ν,α,σ. The ν,α functions also appear in the barotropic variational formalism [1]; however, the σ Lagrange multiplier is of course unique to non-barotropic flows and also appears in non-barotropic magnetohydrodynamics [13]. Variation with respect to ν,α,σ will obviously lead to:

(41) $$\begin{array}{ccc} & & \frac{\partial \rho }{\partial t}+\vec{\nabla }\cdot \left(\rho \vec{v}\right)=0,\\ & & \rho \frac{d\beta }{dt}=0,\\ & & \rho \frac{ds}{dt}=0. \end{array}$$

For the case that ρ is not zero, these are just the mass conservation Equation (1) and the fact that β and *s* are comoving; that is they do not change for any flow element. Varying the action with respect to $\vec{v}$ yields:

(42) $$\delta_{\vec{v}}A=\int d^{3}xdt\rho \delta \vec{v}\cdot \left[\vec{v}-\vec{\nabla }\nu -\alpha \vec{\nabla }\beta -\sigma \vec{\nabla }s\right]+\oint d\vec{A}\cdot \delta \vec{v}\rho \nu +\int d\vec{\Sigma }\cdot \delta \vec{v}\rho \left[\nu \right].$$

The boundary terms above include an integration over the external boundary $\oint d\vec{A}$ and an integral over a cut $\int d\vec{\Sigma }$ that must be introduced when ν is not single-valued, which will be discussed in later sections. The external boundary term becomes zero in cases of astrophysical flows where ρ=0 on the free flow boundary, or when the fluid is contained in a vessel with a no-flux boundary condition $\delta \vec{v}\cdot \hat{n}=0$ (where $\hat{n}$ is a unit vector normal to the boundary). The cut “boundary” term also vanishes if the velocity field only varies parallel to the cut, satisfying a Kutta-type condition. If these boundary terms are zero, $\vec{v}$ is of the following form:

(43) $$\vec{v}=\hat{\vec{v}}\equiv \alpha \vec{\nabla }\beta +\sigma \vec{\nabla }s+\vec{\nabla }\nu .$$

This is a generalized Clebsch representation of the velocity (see for example [6,9,10,14] (p. 248)). We now vary the action with respect to ρ:

(44) $$\begin{array}{ccc} \delta_{\rho }A & = & \int d^{3}xdt\delta \rho \left[\frac{1}{2}{\vec{v}}^{2}-w-\Psi -\frac{\partial \nu }{\partial t}-\vec{v}\cdot \vec{\nabla }\nu \right]\\ & + & \oint d\vec{A}\cdot \vec{v}\delta \rho \nu +\int d\vec{\Sigma }\cdot \vec{v}\delta \rho \left[\nu \right]+\int d^{3}{x\nu \delta \rho |}_{t_{0}}^{t_{1}}. \end{array}$$

Thus if δρ vanishes on the domain’s boundary, on the cut, and at both the initial and final times we obtain:

(45) $$\frac{d\nu }{dt}=\frac{1}{2}{\vec{v}}^{2}-w-\Psi .$$

Let us vary the action with respect to *s*:

(46) $$\begin{array}{ccc} \delta_{s}A & = & \int d^{3}xdt\delta s\left[\frac{\partial \left(\rho \sigma \right)}{\partial t}+\vec{\nabla }\cdot \left(\rho \sigma \vec{v}\right)-\rho T\right]+\int dt\oint d\vec{A}\cdot \rho \sigma \vec{v}\delta s\\ & - & \int d^{3}{x\rho \sigma \delta s|}_{t_{0}}^{t_{1}}, \end{array}$$

in which the temperature is $T=\frac{\partial \varepsilon }{\partial s}$, as defined in Equation (12). According to Equation (43) σ is single-valued; thus no cuts are needed. Using Equation (41) we obtain the following for ρ≠0 locations:

(47) $$\frac{d\sigma }{dt}=T,$$

if we require that $\delta_{s}A=0$ for arbitrary δs.

Finally we vary *A* with respect to β:

(48) $$\begin{array}{ccc} \delta_{\beta }A & = & \int d^{3}xdt\delta \beta \left[\frac{\partial \left(\rho \alpha \right)}{\partial t}+\vec{\nabla }\cdot \left(\rho \alpha \vec{v}\right)\right]\\ & - & \oint d\vec{A}\cdot \vec{v}\rho \alpha \delta \beta -\int d\vec{\Sigma }\cdot \vec{v}\rho \alpha \left[\delta \beta \right]-\int d^{3}{x\rho \alpha \delta \beta |}_{t_{0}}^{t_{1}}. \end{array}$$

Thus if δβ is chosen such that both temporal and spatial boundary terms are null (δβ to be continuous on the cut if it is needed),

(49) $$\frac{\partial \left(\rho \alpha \right)}{\partial t}+\vec{\nabla }\cdot \left(\rho \alpha \vec{v}\right)=0.$$

Using the mass conservation Equation (1), we simply have

(50) $$\rho \frac{d\alpha }{dt}=0.$$

Hence for every location in which ρ≠0, both α and β are comoving coordinates. The vorticity can be derived from Equation (43):

(51) $$\vec{\omega }=\vec{\nabla }\times \vec{v}=\vec{\nabla }\alpha \times \vec{\nabla }\beta +\vec{\nabla }\sigma \times \vec{\nabla }s.$$

Calculating $\frac{\partial \vec{\omega }}{\partial t}$, in which ω is defined by Equation (51) and using Equations (41), (47) and (50), will yield Equation (20). We mention that in particular the six Equations (41), (45), (47) and (50) are equivalent to Salmon’s [2] six equations (3.18).

### Summary

In this section we introduce an action principle given in the Eulerian picture of fluid dynamics. This approach involves the introduction of several constraints that are involved in conserving mass, entropy, and particle identity. The constraints are enforced using Lagrange multipliers, which later appear in the formalism as constraints. The variational equations are obtained, but their connection to the Euler equation is left to the next section.

## 6. Euler’s Equations

Although we obtained from the variational principle the continuity and specific entropy conservation Equations (1) and (3), the rest of the variational equations seem unrelated to the Euler equation (2); however, this impression is false.

We shall now demonstrate that a velocity field given in the generalized Clebsch form of Equation (43), in which the dependent variables α,β,ν,σ,s satisfy Equations (41), (45), (47) and (50), satisfies Euler’s equations. We calculate $\vec{v}$’s material derivative applying Equations (47) and (50):

(52) $$\frac{d\vec{v}}{dt}=\frac{d\vec{\nabla }\nu }{dt}+\frac{d\alpha }{dt}\vec{\nabla }\beta +\alpha \frac{d\vec{\nabla }\beta }{dt}+\frac{d\sigma }{dt}\vec{\nabla }s+\sigma \frac{d\vec{\nabla }s}{dt}=\frac{d\vec{\nabla }\nu }{dt}+\alpha \frac{d\vec{\nabla }\beta }{dt}+T\vec{\nabla }s+\sigma \frac{d\vec{\nabla }s}{dt}.$$

It can be easily shown using Equations (41) and (45) that:

(53) $$\begin{array}{ccc} \frac{d\vec{\nabla }\nu }{dt} & = & \vec{\nabla }\frac{d\nu }{dt}-\vec{\nabla }v_{k}\frac{\partial \nu }{\partial x_{k}}=\vec{\nabla }\left(\frac{1}{2}{\vec{v}}^{2}-w-\Psi \right)-\vec{\nabla }v_{k}\frac{\partial \nu }{\partial x_{k}},\\ \frac{d\vec{\nabla }\beta }{dt} & = & \vec{\nabla }\frac{d\beta }{dt}-\vec{\nabla }v_{k}\frac{\partial \beta }{\partial x_{k}}=-\vec{\nabla }v_{k}\frac{\partial \beta }{\partial x_{k}},\\ \frac{d\vec{\nabla }s}{dt} & = & \vec{\nabla }\frac{ds}{dt}-\vec{\nabla }v_{k}\frac{\partial s}{\partial x_{k}}=-\vec{\nabla }v_{k}\frac{\partial s}{\partial x_{k}}. \end{array}$$

In the above $x_{k}$ are Cartesian coordinates and Einstein’s summation convention is utilized. It follows from Equations (52) and (53) that:

(54) $$\begin{array}{ccc} \frac{d\vec{v}}{dt} & = & -\vec{\nabla }v_{k}\left(\frac{\partial \nu }{\partial x_{k}}+\alpha \frac{\partial \beta }{\partial x_{k}}+\sigma \frac{\partial s}{\partial x_{k}}\right)+\vec{\nabla }\left(\frac{1}{2}{\vec{v}}^{2}-w-\Psi \right)+T\vec{\nabla }s\\ & = & -\vec{\nabla }v_{k}v_{k}+\vec{\nabla }\left(\frac{1}{2}{\vec{v}}^{2}-w-\Psi \right)+T\vec{\nabla }s=-\frac{\vec{\nabla }p}{\rho }-\vec{\nabla }\Psi , \end{array}$$

in which we have used both Equations (19) and (43). Thus Euler’s equations are derived from the action (40) and thus all non-barotropic ideal fluid dynamics equations can be derived from the same action.

### Summary

In this section we show how the Euler equation can be derived from the variational equations.

## 7. Simplified Eulerian Action

The reader might feel that the authors have unnecessarily complicated the theory of fluid dynamics by introducing four additional functions—α,β,ν,σ—alongside the standard variables $\vec{v},\rho ,s$. However, we will demonstrate that this is not the case. The action given in Equation (40) can indeed be simplified with respect to the number of unknown functions, making it more suitable for a pedagogical presentation. It is straightforward to show that the Lagrangian density in Equation (40) can be expressed as follows:

(55) $$\begin{array}{ccc} L & = & -\rho \left[\frac{\partial \nu }{\partial t}+\alpha \frac{\partial \beta }{\partial t}+\sigma \frac{\partial s}{\partial t}+\varepsilon \left(\rho ,s\right)+\Psi \right]+\frac{1}{2}\rho \left[{\left(\vec{v}-\hat{\vec{v}}\right)}^{2}-{\hat{\vec{v}}}^{2}\right]\\ & + & \frac{\partial \left(\nu \rho \right)}{\partial t}+\vec{\nabla }\cdot \left(\nu \rho \vec{v}\right), \end{array}$$

$\hat{\vec{v}}$ stands for $\vec{\nabla }\nu +\alpha \vec{\nabla }\beta +\sigma \vec{\nabla }s$ (see Equation (43)). Thus L has three components:

(56) $$\begin{array}{ccc} L & = & \hat{L}+L_{\vec{v}}+L_{boundary},\\ \hat{L} & \equiv & -\rho [\frac{\partial \nu }{\partial t}+\alpha \frac{\partial \beta }{\partial t}+\sigma \frac{\partial s}{\partial t}+\varepsilon \left(\rho ,s\right)+\Psi +\frac{1}{2}{\left(\vec{\nabla }\nu +\alpha \vec{\nabla }\beta +\sigma \vec{\nabla }s\right)}^{2}],\\ L_{\vec{v}} & \equiv & \frac{1}{2}\rho {\left(\vec{v}-\hat{\vec{v}}\right)}^{2},\\ L_{boundary} & \equiv & \frac{\partial \left(\nu \rho \right)}{\partial t}+\vec{\nabla }\cdot \left(\nu \rho \vec{v}\right). \end{array}$$

The term $L_{\vec{v}}$ is the only one involving $\vec{v}$, and it can be shown that this term will lead to the form given in Equation (43) when the variational derivative is set to zero, without affecting other variational derivatives. It is important to note that the term $L_{boundary}$ consists only of complete partial derivatives, so it does not contribute to the equations but can influence the boundary conditions. Thus, Equations (41), (45), (47) and (50) can be derived using the Lagrangian density $\hat{L}$, where $\hat{\vec{v}}$ replaces $\vec{v}$ in the relevant equations. After solving the six Equations (41), (45), (47) and (50), the functions α,β,ν,σ,s can be substituted into Equation (43) to obtain the physical velocity $\vec{v}$. This approach means that the general non-barotropic fluid dynamics problem can be approached by solving an alternative set of six equations, which can be derived from the Lagrangian density $\hat{L}$, instead of the usual five Equations (1)–(3). The potential Ψ influences the flow dynamics through Equation (45), while thermodynamics impacts flow dynamics through two equations: (45), which depends on the specific enthalpy *w*, and (47), which depends on the temperature *T*. We mention that a variation principle based on $\hat{L}$ was already described by Salmon ([2], Equation (3.16)).

### Summary

In this section we show how the variational variables can be reduced in number from nine to six; this involves taking the velocity field to be a dependent quantity whose value is determined only after the six relevant equations are solved. This is similar to the procedure used in classical electrodynamics, in which the variational principle is also formulated in terms of potentials.

## 8. The Simplified Hamiltonian Formalism

Let us derive the conjugate momenta of the variables appearing in the Lagrangian density $\hat{L}$ defined in Equation (56). A simple calculation will yield

(57) $$\pi_{\nu }\equiv \frac{\partial \hat{L}}{\partial \left(\frac{\partial \nu }{\partial t}\right)}=-\rho ,\;\pi_{\beta }\equiv \frac{\partial \hat{L}}{\partial \left(\frac{\partial \beta }{\partial t}\right)}=-\rho \alpha ,\;\pi_{s}\equiv \frac{\partial \hat{L}}{\partial \left(\frac{\partial s}{\partial t}\right)}=-\rho \sigma .$$

The rest of the canonical momenta $\pi_{\rho },\pi_{\alpha },\pi_{\sigma }$ are null. It thus seems that the six functions appearing in the Lagrangian density $\hat{L}$ can be divided into “approximate” conjugate pairs: (ν,ρ),(α,β),(s,σ). The Hamiltonian density $\hat{H}$ can now be calculated as follows:

(58) $$\hat{H}=\pi_{\nu }\frac{\partial \nu }{\partial t}+\pi_{\beta }\frac{\partial \beta }{\partial t}+\pi_{s}\frac{\partial s}{\partial t}-\hat{L}=\rho \left[\frac{1}{2}{\hat{\vec{v}}}^{2}+\varepsilon \left(\rho ,s\right)+\Psi \right],$$

in which $\hat{\vec{v}}$ is defined in Equation (43). This Hamiltonian density implies the Hamiltonian

(59) $$H=\int d^{3}x\hat{H}=\int d^{3}x\rho \left[\frac{1}{2}{\hat{\vec{v}}}^{2}+\varepsilon \left(\rho ,s\right)+\Psi \right],$$

which is numerically identical to the Hamiltonian introduced by Salmon ([2], (2.18)). However, in Salmon’s case the Hamiltonian is described in terms of a Lagrangian description of the fluid, while in our case the Hamiltonian density (and hence the Hamiltonian) are described by Eulerian variables. Hamilton’s equation would be the same six Equations (41), (45), (47) and (50).

### Summary

The Eulerian action principle in its reduced form allows us to calculate conjugate momenta and thus introduce a Hamiltonian density and finally a Hamiltonian. The Hamiltonian equations are, nevertheless, the same as the equations obtained before.

## 9. Stationary Fluid Dynamics

Stationary flows are a distinctive feature of Eulerian fluid dynamics, which do not have an equivalent in Lagrangian fluid dynamics. In stationary flows, the physical fields $\vec{v},\rho ,s$ remain constant over time (but not necessarily over space). However, this constancy does not mean that the stationary potentials α,β,ν,σ are solely functions of spatial coordinates. In fact, if we were to assume that these potentials depend only on spatial coordinates, it would lead to incorrect conclusions, as the stationary equations of motion could not be derived from the Lagrangian density $\hat{L}$ provided in Equation (56). To resolve this issue, we can proceed as follows: we should let α,ν,σ depend only on the spatial coordinates, while we select β in a specific manner:

(60) $$\beta =\bar{\beta }-t,$$

$\bar{\beta }$ depends only on the spatial coordinates. The Lagrangian density $\hat{L}$ of Equation (56) is thus:

(61) $$\hat{L}=\rho \left(\alpha -\varepsilon \left(\rho ,s\right)-\Psi -\frac{1}{2}{\left(\vec{\nabla }\nu +\alpha \vec{\nabla }\beta +\sigma \vec{\nabla }s\right)}^{2}\right).$$

Varying $\hat{L}=\int \hat{L}d^{3}x$ with respect to α,β,ν,ρ,σ,s and equating the variation in the action to null (assuming the relevant spatial and temporal boundary conditions) leads to a set of equations:

(62) $$\begin{array}{ccc} & & \vec{\nabla }\cdot \left(\rho \hat{\vec{v}}\right)=0,\\ & & \rho \hat{\vec{v}}\cdot \vec{\nabla }\alpha =0,\\ & & \rho (\hat{\vec{v}}\cdot \vec{\nabla }\bar{\beta }-1)=0,\\ & & \alpha =\frac{1}{2}{\hat{\vec{v}}}^{2}+w+\Psi ,\\ & & \rho \hat{\vec{v}}\cdot \vec{\nabla }s=0,\\ & & \rho \hat{\vec{v}}\cdot \vec{\nabla }\sigma =\rho T, \end{array}$$

which simplifies for any spatial point in which the fluid density is not null to

(63) $$\begin{array}{ccc} & & \vec{\nabla }\cdot \left(\rho \hat{\vec{v}}\right)=0,\\ & & \hat{\vec{v}}\cdot \vec{\nabla }\alpha =0,\\ & & \hat{\vec{v}}\cdot \vec{\nabla }\bar{\beta }=1,\\ & & \alpha =\frac{1}{2}{\hat{\vec{v}}}^{2}+w+\Psi ,\\ & & \hat{\vec{v}}\cdot \vec{\nabla }s=0,\\ & & \hat{\vec{v}}\cdot \vec{\nabla }\sigma =T. \end{array}$$

α is just the constant suggested by Bernoulli. Calculations essentially identical to the ones performed previously show that the above equations are equivalent to stationary Euler equations:

(64) $$\left(\vec{v}\cdot \vec{\nabla }\right)\vec{v}=-\frac{\vec{\nabla }p}{\rho }-\vec{\nabla }\Psi .$$

### Summary

The Eulerian action principle in its reduced form is considered for the special case of stationary flows. It is emphasized that a stationary flow field does not imply stationary Clebsch potentials, but rather a very specific time dependency, as described above.

## 10. Constants of Motion

A well-known concept in fluid dynamics is circulation around a trajectory that is comoving with the flow:

(65) $$C\equiv \oint \vec{v}\cdot d\vec{l},$$

$d\vec{l}$ is an infinitesimal length oriented along the trajectory. It follows from Equation (54) that

(66) $$\frac{dC}{dt}=\oint T\vec{\nabla }s\cdot d\vec{l}=\oint Tds.$$

Hence circulation is not conserved unless the flow is barotropic (which means a uniform specific entropy throughout the flow) or the trajectory happens to be on a constant-specific-entropy or constant-temperature surface. However, using the variational equations that we obtained in the previous sections we can generalize circulation conservation to an arbitrary comoving trajectory as follows. First we define a topological velocity field:

(67) $${\vec{v}}_{t}\equiv \vec{v}-\sigma \vec{\nabla }s,$$

σ and *s* are defined in previous sections. Next we define the circulation of the topological flow field in an analogous way to the definition of standard circulation:

(68) $$C_{t}\equiv \oint {\vec{v}}_{t}\cdot d\vec{l}=C-\oint \sigma \vec{\nabla }s\cdot d\vec{l}=C-\oint \sigma ds.$$

It is easy to show that this quantity is conserved for any trajectory:

(69) $$\frac{dC_{t}}{dt}=\frac{dC}{dt}-\oint \frac{d\sigma }{dt}ds=\oint Tds-\oint Tds=0.$$

in which we have used Equations (47) and (66). Taking into account the generalized Clebsch representation Equation (43), it follows that:

(70) $${\vec{v}}_{t}=\vec{v}-\sigma \vec{\nabla }s=\alpha \vec{\nabla }\beta +\vec{\nabla }\nu .$$

Hence the topological flow field has a standard Clebsch form. Moreover, the associated vorticity of this flow is:

(71) $${\vec{\omega }}_{t}=\vec{\nabla }\times {\vec{v}}_{t}=\vec{\nabla }\alpha \times \vec{\nabla }\beta =\vec{\omega }-\vec{\nabla }\sigma \times \vec{\nabla }s.$$

We can use Stokes’ theorem to write the topological circulation $C_{t}$ as the topological vorticity flux $\Phi_{t}$ through a comoving surface:

(72) $$C_{t}=\oint {\vec{v}}_{t}\cdot d\vec{l}=\int \vec{\nabla }\times {\vec{v}}_{t}\cdot d\vec{A}=\int {\vec{\omega }}_{t}\cdot d\vec{A}=\int \vec{\nabla }\alpha \times \vec{\nabla }\beta \cdot d\vec{A}=\int d\alpha d\beta =\Phi_{t}.$$

Hence a tube of topological vorticity lines will not change its flux due to the flow dynamics (see also [15]). This remains true even if the tube’s cross-section is infinitesimal, implying that each vortex line is comoving and thus the topology of the topological vortex lines is conserved as well, and they cannot be unknotted by the flow if they are initially knotted. This is of course the reason why they are called topological in the first place. Using Equations (41) and (50), a straightforward calculation yields:

(73) $$\frac{\partial {\vec{\omega }}_{t}}{\partial t}=\vec{\nabla }\times \left(\vec{v}\times {\vec{\omega }}_{t}\right),$$

which is a sufficient condition for the topological vortex line to comove with $\vec{v}$. In barotropic fluid dynamics the helicity is a (topological) constant of motion:

(74) $$H\equiv \int \vec{\omega }\cdot \vec{v}d^{3}x,$$

which is a measure of the knottiness of lines for $\vec{\omega }$ [5]. However, this quantity is not constant in non-barotropic flows. Nevertheless, the previous discussion shows that this quantity can be easily generalized using the concept of topological flow fields and their topological vorticity:

(75) $$H_{t}\equiv \int {\vec{\omega }}_{t}\cdot {\vec{v}}_{t}d^{3}x.$$

A straightforward calculation will show that this is indeed conserved (see also [15]). We write Equation (75) in terms of the potentials α,β,ν; the scalar product ${\vec{\omega }}_{t}\cdot {\vec{v}}_{t}$ is

(76) $${\vec{\omega }}_{t}\cdot {\vec{v}}_{t}=\left(\vec{\nabla }\alpha \times \vec{\nabla }\beta \right)\cdot \vec{\nabla }\nu .$$

**Figure 1:**
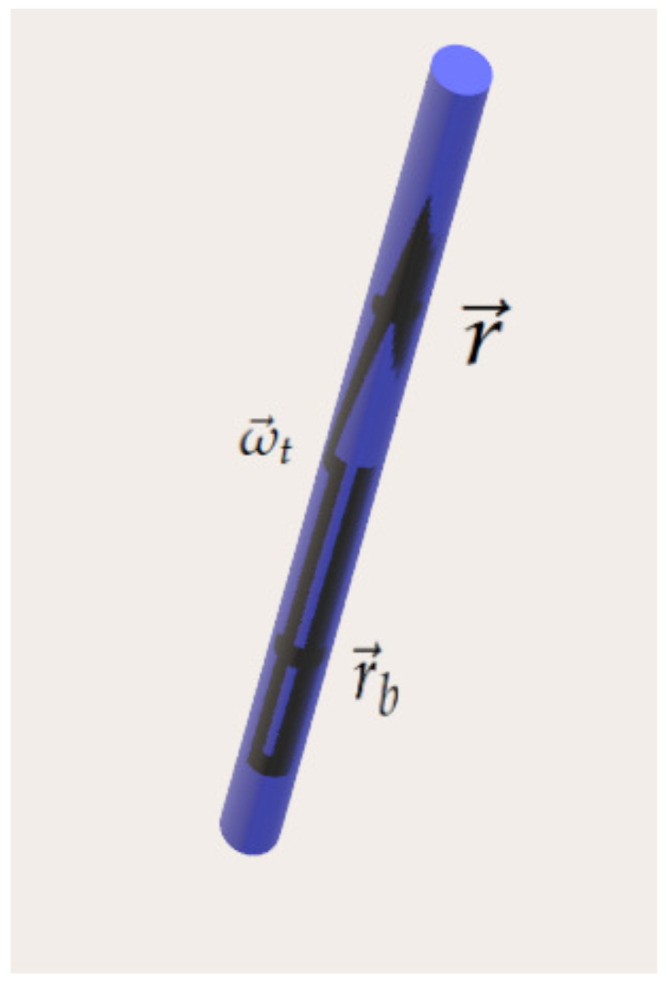
A topological vortex tube.

We introduce a local vector basis: $\left(\vec{\nabla }\alpha ,\vec{\nabla }\beta ,\vec{\nabla }\mu \right)$, in which μ is a comoving function with a gradient that is not parallel to either $\vec{\nabla }\alpha$ or $\vec{\nabla }\beta$. The fact that α and β are comoving is known from Equations (41) and (50). The metage μ was defined in [16] for barotropic flows, but here we extend the definition for the non-barotropic case using the concept of topological vortex flux, which is equivalent to topological circulation. We consider a thin topological vortex tube surrounding a topological vorticity line, as in Figure 1.

Let us select two comoving points along this line (basically two fluid element locations), which we denote by ${\vec{r}}_{b}$ and $\vec{r}$, defining a section along the tube. We shall define the vorticity line metage as the mass *M* in this section of the thin tube divided by the topological vortex flux carried by the same thin tube:

(77) $$\mu \left(\vec{r}\right)=\frac{M}{\Phi_{t}},$$

in which we notice that since this is a topological vortex tube, $\Phi_{t}$ does not depend on the specific location along the tube in which it is calculated. This is obviously a conserved quantity as both mass in the comoving tube and topological circulation are conserved by the motion. Thus,

(78) $$\frac{d\mu }{dt}=0\;.$$

Now if we take the cross-section area of the tube that is perpendicular to the topological vortex vector to be dS, it follows that the mass in this tube is:

(79) $$M=\int_{{\vec{r}}_{b}}^{\vec{r}}\rho dSdl,$$

and the vortex flux is given by:

(80) $$\Phi_{t}=\omega_{t}dS.$$

Thus we obtain:

(81) $$\mu \left(\vec{r}\right)=\int_{{\vec{r}}_{b}}^{\vec{r}}\frac{\rho dSdl}{\omega_{t}dS}=\int_{{\vec{r}}_{b}}^{\vec{r}}\frac{\rho }{\omega_{t}}dl.$$

From this it follows that:

(82) $${\vec{\omega }}_{t}\cdot \vec{\nabla }\mu =\rho \;.$$

Taking into account Equation (71), we arrive at:

(83) $$\rho =\left(\vec{\nabla }\alpha \times \vec{\nabla }\beta \right)\cdot \vec{\nabla }\mu =\frac{\partial (\alpha ,\beta ,\mu )}{\partial (x,y,z)}.$$

Using the α,β,μ functions, we can now write $\vec{\nabla }\nu$ as follows:

(84) $$\vec{\nabla }\nu =\frac{\partial \nu }{\partial \alpha }\vec{\nabla }\alpha +\frac{\partial \nu }{\partial \beta }\vec{\nabla }\beta +\frac{\partial \nu }{\partial \mu }\vec{\nabla }\mu .$$

Thus:

(85) $${\vec{\omega }}_{t}\cdot {\vec{v}}_{t}=\frac{\partial \nu }{\partial \mu }\left(\vec{\nabla }\alpha \times \vec{\nabla }\beta \right)\cdot \vec{\nabla }\mu =\frac{\partial \nu }{\partial \mu }\frac{\partial (\alpha ,\beta ,\mu )}{\partial (x,y,z)}.$$

Inserting Equation (85) into Equation (75), we obtain:

(86) $$H_{t}=\int \frac{\partial \nu }{\partial \mu }d\mu d\alpha d\beta .$$

In certain situations, it might be necessary to divide the flow domain into separate regions or patches, each having different definitions for μ,α,β. This division is not considered a limitation of our formalism, as the topology of the flow remains consistent with the flow equations. In such cases, the quantity $H_{t}$ should be calculated by summing the contributions from each patch. We can envision the fluid domain as being composed of thin, closed tubes of topological vortex lines, each identified by the values of (α,β). Integrating along these thin tubes in the metage direction yields the following result:

(87) $$\oint_{\alpha ,\beta }\frac{\partial \nu }{\partial \mu }d\mu ={\left[\nu \right]}_{\alpha ,\beta },$$

In this context, ${\left[\nu \right]}_{\alpha ,\beta }$ represents the discontinuity of the function ν along its cut. This means that if a thin tube of vortex lines exists where ν is single-valued, it does not contribute to the topological helicity integral. When we substitute the expression from the equation $\nu_{\mathrm{cut}}$ into the helicity equation, we obtain the following result:

(88) $$H_{t}=\int {\left[\nu \right]}_{\alpha ,\beta }d\alpha d\beta =\int \left[\nu \right]d\Phi_{t},$$

in which Equation (68) is used. Thus:

(89) $$\left[\nu \right]=\frac{dH_{t}}{d\Phi_{t}},$$

The discontinuity of ν represents the density of topological helicity per unit of topological vortex flux within a tube. This means that the Clebsch representation does not imply zero topological helicity; instead, it can accommodate non-zero topological helicity, as demonstrated above. Moreover, according to Equation (45):

(90) $$\frac{d[\nu ]}{dt}=0.$$

We can conclude not only that topological helicity is conserved as an overall quantity across the entire flow domain, but also that the local density of topological helicity per unit of topological vortex flux remains conserved.

It is known from the work of Tur and Yanovsky [17] and Sagdeev et al. [18] on comoving invariants in MHD and in ideal fluids that many more such constants of topological significance exist. In fact they derived infinite hierarchies of conservation laws associated with fluid relabeling symmetries; however, the variational variables allow a particular simple and elegant form of the topological circulation and helicity invariants.

### Summary

We show how the variational variables are useful for generalizing well-known conservation laws of ideal barotropic flows, such as the conservation of circulation and helicity.

## 11. A Simpler Variational Principle of Non-Stationary Fluid Dynamics

Lynden-Bell and Katz [16] demonstrated that an Eulerian variational principle for non-stationary barotropic fluid dynamics could be expressed using just two functions: the density (ρ) and the load (λ). However, the velocity was implicitly defined through a partial differential equation, and its variations were constrained by this equation. This approach has been similarly criticized in their work on non-barotropic flows [19]. However, Yahalom & Lynden-Bell [1] have shown that a true variational principle (which is unconstrained and without implicit definitions) for barotropic flows can be formulated in terms of three dependent variables ρ,ν and an additional comoving dependent variable λ. Here we show that for non-barotropic flows four dependent variables will suffice; these are ρ,ν,s and an additional comoving function.

Consider the last two equations in (41) and write them explicitly in terms of the generalized Clebsch form defined in Equation (43):

(91) $$\begin{array}{ccc} & & \frac{d\beta }{dt}=\frac{\partial \beta }{\partial t}+\vec{v}\cdot \vec{\nabla }\beta =\frac{\partial \beta }{\partial t}+\left(\alpha \vec{\nabla }\beta +\sigma \vec{\nabla }s+\vec{\nabla }\nu \right)\cdot \vec{\nabla }\beta =0,\\ & & \frac{ds}{dt}=\frac{\partial s}{\partial t}+\vec{v}\cdot \vec{\nabla }s=\frac{\partial s}{\partial t}+\left(\alpha \vec{\nabla }\beta +\sigma \vec{\nabla }s+\vec{\nabla }\nu \right)\cdot \vec{\nabla }s=0. \end{array}$$

We thus derive two algebraic equations for the variables α,σ that can be readily solved. Introducing the notation:

(92) $$\begin{array}{ccc} & & a_{\beta \beta }={\left(\vec{\nabla }\beta \right)}^{2},\;a_{ss}={\left(\vec{\nabla }s\right)}^{2},\;a_{s\beta }=a_{\beta s}=\vec{\nabla }\beta \cdot \vec{\nabla }s,\\ & & k_{\beta }=-\frac{\partial \beta }{\partial t}-\vec{\nabla }\nu \cdot \vec{\nabla }\beta ,\;k_{s}=-\frac{\partial s}{\partial t}-\vec{\nabla }\nu \cdot \vec{\nabla }s. \end{array}$$

we obtain both α,σ as functionals of the variables s,β,ν:

(93) $$\alpha \left[s,\beta ,\nu \right]=\frac{a_{ss}k_{\beta }-a_{s\beta }k_{s}}{a_{ss}a_{\beta \beta }-a_{s\beta }^{2}},\;\sigma \left[s,\beta ,\nu \right]=\frac{a_{\beta \beta }k_{s}-a_{s\beta }k_{\beta }}{a_{ss}a_{\beta \beta }-a_{s\beta }^{2}}.$$

In Hamiltonian language this means that the canonical momenta of β and *s* are now given expressions of (s,β,ν):

(94) $$\pi_{\beta }=-\frac{1}{\rho }\left(\frac{a_{ss}k_{\beta }-a_{s\beta }k_{s}}{a_{ss}a_{\beta \beta }-a_{s\beta }^{2}}\right),\;\pi_{s}=-\frac{1}{\rho }\left(\frac{a_{\beta \beta }k_{s}-a_{s\beta }k_{\beta }}{a_{ss}a_{\beta \beta }-a_{s\beta }^{2}}\right).$$

Similarly the velocity field is now a functional of the three variables s,β,ν:

(95) $$\vec{v}\left[s,\beta ,\nu \right]=\alpha \left[s,\beta ,\nu \right]\vec{\nabla }\beta +\sigma \left[s,\beta ,\nu \right]\vec{\nabla }s+\vec{\nabla }\nu .$$

Finally the Lagrangian density is a functional of the four variables s,β,ν,ρ:

(96) $$\begin{array}{ccc} \hat{L}\left[s,\beta ,\nu ,\rho \right] & = & -\rho \left[\frac{\partial \nu }{\partial t}+\alpha \left[s,\beta ,\nu \right]\frac{\partial \beta }{\partial t}+\sigma \left[s,\beta ,\nu \right]\frac{\partial s}{\partial t}+\varepsilon \left(\rho ,s\right)+\Psi \right.\\ & + & \left.\frac{1}{2}{\left(\vec{\nabla }\nu +\alpha \left[s,\beta ,\nu \right]\vec{\nabla }\beta +\sigma \left[s,\beta ,\nu \right]\vec{\nabla }s\right)}^{2}\right]. \end{array}$$

the variation in which will lead to the following four equations, which replace the original set of Equations (1)–(3):

(97) $$\frac{\partial \rho }{\partial t}+\vec{\nabla }\cdot \left(\rho \vec{v}\right)=0,\;\frac{d\nu }{dt}=\frac{1}{2}{\vec{v}}^{2}-w-\Psi ,\;\frac{d\sigma [s,\beta ,\nu ]}{dt}=T,\;\frac{d\alpha [s,\beta ,\nu ]}{dt}=0.$$

Written explicitly the form of these equations may look rather complicated.

### Summary

We show how to reduce the variational formalism even further. Using specific variables for which the variational equations can be solved algebraically, we reduce the dynamical degrees of freedom from six to four variables.

## 12. Example: A Flow Solution in Circular Toroidal Coordinates

Consider a stationary fluid where both the velocity and vorticity lines are confined to toroidal surfaces, defined as surfaces where the radial coordinate $\bar{r}$ remains constant. The cylindrical polar coordinates (R,ϕ,z) are used to describe the position in space, with $\bar{r}$ being a function that defines these toroidal surfaces:

(98) $$\bar{r}\equiv \sqrt{z^{2}+{\left(R-1\right)}^{2}}.$$

**Figure 2:**
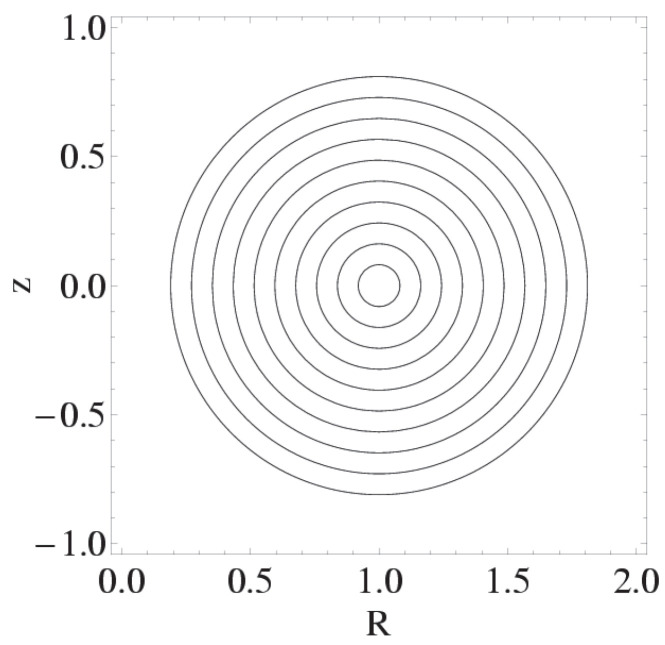
*R*-*z* cross-section of the nested tori, with centre at R=1,z=0.

A visual representation of nested tori is depicted in Figure 2. This cross-section shows how these toroidal surfaces are layered, with each torus having a distinct but constant $\bar{r}$, forming a series of concentric structures. These nested tori are the loci of velocity and vorticity lines, which are confined to specific toroidal surfaces.

As the velocity field lines are constrained to these tori, we have according to Equation (63) $\alpha =\alpha (\bar{r})$. For a uniform specific entropy *s*, it follows from Equation (51) that the vorticity lines are also constrained to the same tori.

We assume that the flow is confined between two specific tori, bounded by $\epsilon \leq \bar{r}\leq a$, where *a* and ϵ are constants and satisfy 0<ϵ<a<1. Within this range, we introduce an angular coordinate η on the tori. This coordinate will help describe the movement along the surface of the tori, providing a clearer understanding of the flow’s structure on these confined surfaces:

(99) $$\eta \equiv \arctan\frac{z}{R-1}.$$

In this scenario, we establish an orthogonal coordinate system on the toroidal surfaces using the coordinates $\bar{r},\phi ,\eta$, where $\bar{r}$ represents the radial coordinate, ϕ is the azimuthal angle, and η is the angular coordinate introduced above. These coordinates are orthogonal to one another, which simplifies the application of standard vector calculus operations in terms of these toroidal coordinates. The mathematical operators used in vector analysis, such as the gradient, divergence, and curl, are given below to facilitate analysis in this specific coordinate system:

(100) $$\vec{\nabla }f=\hat{\bar{r}}\frac{\partial f}{\partial \bar{r}}+\hat{\phi }\frac{1}{R}\frac{\partial f}{\partial \phi }+\hat{\eta }\frac{1}{\bar{r}}\frac{\partial f}{\partial \eta },$$

(101) $$\vec{\nabla }\cdot \vec{A}=\frac{1}{\bar{r}R}\left(\frac{\partial (\bar{r}RA_{\bar{r}})}{\partial \bar{r}}+\frac{\partial (\bar{r}A_{\phi })}{\partial \phi }+\frac{\partial (RA_{\eta })}{\partial \eta }\right),$$

(102) $${\vec{\nabla }}^{2}f=\frac{1}{\bar{r}R}\left(\frac{\partial }{\partial \bar{r}}\left(\bar{r}R\frac{\partial f}{\partial \bar{r}}\right)+\frac{\partial }{\partial \phi }\left(\frac{\bar{r}}{R}\frac{\partial f}{\partial \phi }\right)+\frac{\partial }{\partial \eta }\left(\frac{R}{\bar{r}}\frac{\partial f}{\partial \eta }\right)\right),$$

(103) $$\begin{array}{ccc} \vec{\nabla }\times \vec{A} & = & \frac{1}{\bar{r}R}\left[\frac{\partial (\bar{r}A_{\eta })}{\partial \phi }-\frac{\partial (RA_{\phi })}{\partial \eta }\right]\hat{\bar{r}}+\frac{1}{\bar{r}}\left[\frac{\partial (A_{\bar{r}})}{\partial \eta }-\frac{\partial (\bar{r}A_{\eta })}{\partial \bar{r}}\right]\hat{\phi }\\ & + & \frac{1}{R}\left[\frac{\partial (RA_{\phi })}{\partial \bar{r}}-\frac{\partial (A_{\bar{r}})}{\partial \phi }\right]\hat{\eta }. \end{array}$$

### 12.1. The Toroidal Velocity Field

According to the equation represented by Equation (43), we can express the velocity field $\vec{v}$ in a specific form. This equation allows us to rewrite the velocity in a way that reflects the underlying physical principles or constraints imposed by the fluid’s motion. The equation provides a structured relationship between the velocity and other relevant variables, ensuring consistency with the fluid dynamics framework being considered. The form of $\vec{v}$ in Equation (43) plays a central role in analyzing and solving fluid dynamics problems in this context:

(104) $$\vec{v}=\alpha \vec{\nabla }\beta +\vec{\nabla }\nu =\vec{\nabla }\left(\alpha \beta +\nu \right)-\beta \vec{\nabla }\alpha .$$

Introducing the definition below allows us to establish a simpler mathematical and physical concept:

(105) $$\tilde{\nu }=\alpha \beta +\nu ,$$

In this context, Equation (104) is transformed into a new expression that conforms to the established toroidal coordinate system. This step adjusts the general velocity form to be compatible with the specific geometry of the system, which involves toroidal surfaces. By translating the velocity components into the coordinates of the toroidal framework—typically involving $\bar{r}$, ϕ, and η—we adapt the original equation to reflect the structure of the flow on these nested tori. This adaptation is essential for analyzing the dynamics of flows constrained within such surfaces:

(106) $$\vec{v}=\vec{\nabla }\tilde{\nu }-\beta \vec{\nabla }\alpha .$$

Since the velocity vector $\vec{v}$ is assumed to be confined to the toroidal surfaces, we have certain conditions that simplify the analysis. Specifically, the components of the gradient of α along the directions of $\hat{\eta }$ and $\hat{\phi }$ are both zero. Also the component of the velocity along the $\hat{\bar{r}}$ direction, ${\vec{v}}_{\bar{r}}=\vec{v}\cdot \hat{\bar{r}}$, is also zero. This leads to the following simplified expression, represented by a partial derivative notation $\partial f/\partial x\equiv \partial_{x}f$:

(107) $$\partial_{\bar{r}}\tilde{\nu }=\beta \partial_{\bar{r}}\alpha ,\;v_{\eta }=\frac{1}{\bar{r}}\partial_{\eta }\tilde{\nu }.\;v_{\phi }=\frac{1}{R}\partial_{\phi }\tilde{\nu }.$$

Provided that $\tilde{\nu }$ exists it follows that under smoothness assumptions we obtain the equality:

(108) $$\partial_{\eta \phi }^{2}\tilde{\nu }=\partial_{\phi \eta }^{2}\tilde{\nu }.$$

It thus follows that:

(109) $$\partial_{\phi }\left(\bar{r}v_{\eta }\right)=\partial_{\eta }\left(Rv_{\phi }\right).$$

This equation has the following solution:

(110) $$v_{\eta }=\frac{\Gamma_{S}\left(\bar{r}\right)}{2\pi \bar{r}},\;v_{\phi }=\frac{\Gamma_{L}\left(\bar{r},\phi \right)}{2\pi R}.$$

As $\rho \vec{v}$ is a divergence-less vector according to Equation (63) and since is must also be orthogonal to $\vec{\nabla }\alpha$, it follows that a function μ exists such that:

(111) $$\vec{v}=\frac{\vec{\nabla }\mu \times \vec{\nabla }\alpha }{\rho }.$$

It can be shown that this function can be chosen to be a metage function as defined in Equation (77). In the current case we have:

(112) $$v_{\eta }=-\frac{\partial_{\bar{r}}\alpha }{\rho R}\partial_{\phi }\mu ,\;v_{\phi }=\frac{\partial_{\bar{r}}\alpha }{\rho \bar{r}}\partial_{\eta }\mu .$$

If the function μ indeed exists and is smooth, we have:

(113) $$\partial_{\eta \phi }^{2}\mu =\partial_{\phi \eta }^{2}\mu .$$

And thus:

(114) $$\partial_{\phi }\left(\frac{\rho \bar{r}v_{\phi }}{\partial_{\bar{r}}\alpha }\right)+\partial_{\eta }\left(\frac{\rho Rv_{\eta }}{\partial_{\bar{r}}\alpha }\right)=0,$$

which implies:

(115) $$\partial_{\phi }\left(\rho \bar{r}v_{\phi }\right)+\partial_{\eta }\left(\rho Rv_{\eta }\right)=0.$$

Inserting Equation (110) into Equation (115), it follows that:

(116) $$\partial_{\phi }\left(\rho \bar{r}\frac{\Gamma_{L}\left(\bar{r},\phi \right)}{R}\right)+\partial_{\eta }\left(\rho R\frac{\Gamma_{S}\left(\bar{r}\right)}{\bar{r}}\right)=0.$$

Which is simplified to the form below:

(117) $$\frac{\bar{r}}{R}\partial_{\phi }\left(\rho \Gamma_{L}\left(\bar{r},\phi \right)\right)+\frac{\Gamma_{S}\left(\bar{r}\right)}{\bar{r}}\partial_{\eta }\left(\rho R\right)=0.$$

A non-unique solution of the above is given below:

(118) $$\rho =\frac{g(\bar{r})}{R},\;\Gamma_{L}=\Gamma_{L}\left(\bar{r}\right).$$

Thus $\Gamma_{L}$ is the circulation along the “long path”:

(119) $$\oint_{\eta =const.,\bar{r}=const.}\vec{v}\cdot d\vec{s}=\int_{0}^{2\pi }v_{\phi }Rd\phi =2\pi Rv_{\phi }=\Gamma_{L},$$

$\Gamma_{S}$, on the other hand, is the circulation along the “short path”:

(120) $$\oint_{\phi =const.,\bar{r}=const.}\vec{v}\cdot d\vec{s}=\int_{0}^{2\pi }v_{\eta }\bar{r}d\eta =2\pi \bar{r}v_{\eta }=\Gamma_{S}.$$

Thus the velocity field is of the form:

(121) $$v_{\eta }=\frac{\Gamma_{S}\left(\bar{r}\right)}{2\pi \bar{r}},\;v_{\phi }=\frac{\Gamma_{L}\left(\bar{r}\right)}{2\pi R},\;v_{\bar{r}}=0.$$

The vorticity is calculated using the definition Equation (5) with the help of Equation (103):

(122) $$\begin{array}{ccc} \omega_{\bar{r}} & = & 0,\\ \omega_{\eta } & = & \frac{1}{R}\partial_{\bar{r}}\left(Rv_{\phi }\right)=+\frac{1}{2\pi R}\partial_{\bar{r}}\Gamma_{L}\left(\bar{r}\right),\\ \omega_{\phi } & = & -\frac{1}{\bar{r}}\partial_{\bar{r}}\left(\bar{r}v_{\eta }\right)=-\frac{1}{2\pi \bar{r}}\partial_{\bar{r}}\Gamma_{S}\left(\bar{r}\right). \end{array}$$

Solving Equation (112) for μ, we find the expression:

(123) $$\mu =-\frac{g\Gamma_{S}\phi }{2\pi \bar{r}\partial_{\bar{r}}\alpha }+\frac{g\bar{r}\Gamma_{L}}{2\pi \partial_{\bar{r}}\alpha }\int_{0}^{\eta }\frac{d\eta^{\prime }}{{\left(1+\bar{r}\cos\eta^{\prime }\right)}^{2}}+\mu_{2}\left(\bar{r}\right),$$

in which:

(124) $$II\left(\bar{r},\eta \right)\equiv \int_{0}^{\eta }\frac{d\eta^{\prime }}{{\left(1+\bar{r}\cos\eta^{\prime }\right)}^{2}}=\frac{I(\bar{r},\eta )}{1-{\bar{r}}^{2}}-\frac{\bar{r}\sin\eta }{\left(1-{\bar{r}}^{2}\right)\left(1+\bar{r}\cos\eta \right)},$$

and also:

(125) $$\begin{array}{ccc} I(\bar{r},\eta ) & \equiv & \int_{0}^{\eta }\frac{d\eta^{\prime }}{1+\bar{r}\cos\eta^{\prime }}=\frac{2}{\sqrt{1-{\bar{r}}^{2}}}\left[\arctan(\sqrt{\frac{1-\bar{r}}{1+\bar{r}}}\tan\left(\frac{\eta }{2}\right))\right.\\ & + & \left.\left\{\begin{array}{cc} 0, & 0\leq \eta <\pi \\ \pi , & \pi \leq \eta <2\pi \end{array}\right.\right]. \end{array}$$

**Figure 3:**
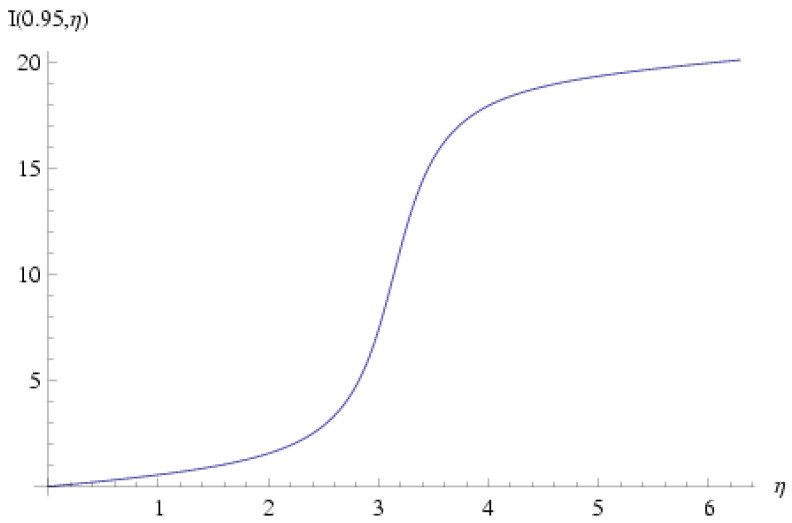
$I(\bar{r},\eta )$ for $\bar{r}=0.95$. This function is defined in Equation (125) and is needed in order to calculate the function II defined in Equation (124) and eventually the metage μ, which is given in Equation (123). It is obvious that this function is multi-valued and does return to its original value after a 2π rotation of η.

**Figure 4:**
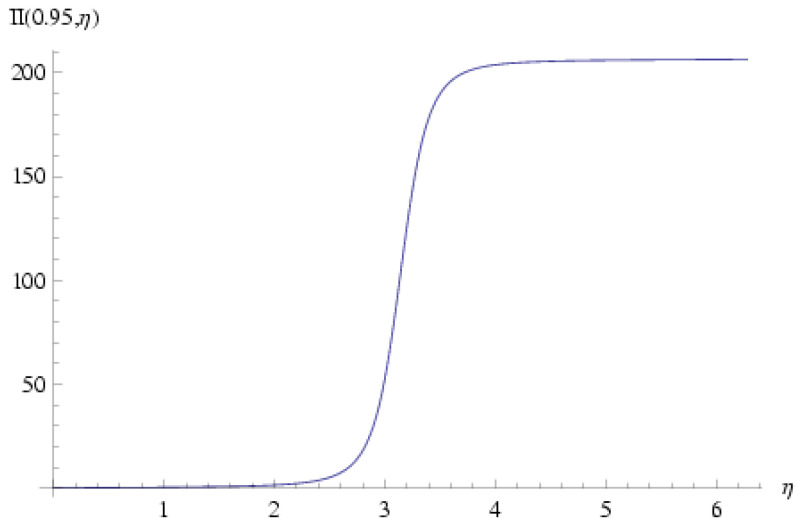
$II(\bar{r},\eta )$ for $\bar{r}=0.95$. This function is defined in Equation (124) and is needed in order to evaluate the metage μ, which is given in Equation (123). It is obvious that this function is multi-valued and does return to its original value after a 2π rotation of η.

Plots of $I(\bar{r},\eta )$ and $II(\bar{r},\eta )$ are shown in Figure 3 and Figure 4 for the representative value $\bar{r}=0.95$; obviously both functions are non-single-valued in η.

Combining Equation (111) with Equation (63) for β, we arrive at the Jacobian equation for ρ:

(126) $$\rho =\vec{\omega }\cdot \vec{\nabla }\mu =\vec{\nabla }\mu \cdot \left(\vec{\nabla }\alpha \times \vec{\nabla }\beta \right)=\frac{\partial (\alpha ,\beta ,\mu )}{\partial (x,y,z)},$$

see also Equation (83). Inserting Equations (122) and (123) into Equation (126), it follows that:

(127) $$\rho =\frac{\omega_{\phi }}{R}\left(-\frac{\rho R\Gamma_{S}}{2\pi \bar{r}\partial_{\bar{r}}\alpha }\right)+\frac{\omega_{\eta }}{\bar{r}}\left(\frac{\rho \bar{r}\Gamma_{L}}{2\pi R\partial_{\bar{r}}\alpha }\right).$$

Eliminating ρ and multiplying both sides by $\partial_{\bar{r}}\alpha$, we obtain:

(128) $$\partial_{\bar{r}}\alpha =\frac{\omega_{\eta }\Gamma_{L}}{2\pi R}-\frac{\omega_{\phi }\Gamma_{S}}{2\pi \bar{r}}.$$

Inserting the vorticity field into the above equation, the following explicit form is derived:

(129) $$\partial_{\bar{r}}\alpha =\frac{\Gamma_{L}\left(\bar{r}\right)\partial_{\bar{r}}\Gamma_{L}\left(\bar{r}\right)}{{\left(2\pi R\right)}^{2}}+\frac{\Gamma_{S}\left(\bar{r}\right)\partial_{\bar{r}}\Gamma_{S}\left(\bar{r}\right)}{{\left(2\pi \bar{r}\right)}^{2}}=\frac{\Gamma_{L}\left(\bar{r}\right)\partial_{\bar{r}}\Gamma_{L}\left(\bar{r}\right)}{{\left(2\pi \left(1+\bar{r}\cos\eta \right)\right)}^{2}}+\frac{\Gamma_{S}\left(\bar{r}\right)\partial_{\bar{r}}\Gamma_{S}\left(\bar{r}\right)}{{\left(2\pi \bar{r}\right)}^{2}}.$$

As α is a function of $\bar{r}$ and not of η, it follows that

(130) $$\partial_{\bar{r}}\Gamma_{L}\left(\bar{r}\right)=0\Rightarrow \Gamma_{L}\left(\bar{r}\right)=\Gamma_{L}=cst.,$$

Thus

(131) $$\partial_{\bar{r}}\alpha =\frac{\Gamma_{S}\left(\bar{r}\right)\partial_{\bar{r}}\Gamma_{S}\left(\bar{r}\right)}{{\left(2\pi \bar{r}\right)}^{2}},\;\omega_{\eta }=0.$$

We conclude that up to a constant factor α is not an arbitrary function but is dictated by the short-way circulation.

We are now in a position to obtain explicit formulae for the rest of the variation functions. From Equation (107) it follows that:

(132) $$\tilde{\nu }=\Gamma_{S}\left(\bar{r}\right)\frac{\eta }{2\pi }+\Gamma_{L}\frac{\phi }{2\pi }+{\tilde{\nu }}_{2}\left(\bar{r}\right),$$

and from Equation (107) for β, we derive:

(133) $$\beta =\frac{\partial_{\bar{r}}\Gamma_{S}}{\partial_{\bar{r}}\alpha }\frac{\eta }{2\pi }+\beta_{2}\left(\bar{r}\right)=\frac{2\pi {\bar{r}}^{2}}{\Gamma_{S}\left(\bar{r}\right)}\eta +\beta_{2}\left(\bar{r}\right).$$

Thus Equation (105) yields an expression for ν:

(134) $$\nu =\tilde{\nu }-\alpha \beta =\left(\Gamma_{S}-\frac{{\left(2\pi \bar{r}\right)}^{2}\alpha }{\Gamma_{S}}\right)\frac{\eta }{2\pi }+\Gamma_{L}\frac{\phi }{2\pi }+\nu_{2}\left(\bar{r}\right).$$

### 12.2. Helicity

Flow helicity can be obtained using Equation (88). ν discontinuity along the the line of fixed α and β is ν’s ϕ discontinuity. It thus follows from Equation (134) that:

(135) $${\left[\nu \right]}_{\phi }=\Gamma_{L}.$$

The vorticity flux element is:

(136) $$d\Phi =\vec{\omega }\cdot d\vec{S}=\omega_{\phi }dS_{\phi }=-\frac{1}{2\pi \bar{r}}\partial_{\bar{r}}\Gamma_{S}\left(\bar{r}\right)\bar{r}d\bar{r}d\eta =-\frac{1}{2\pi }\partial_{\bar{r}}\Gamma_{S}\left(\bar{r}\right)d\bar{r}d\eta .$$

It follows that the helicity can be calculated easily:

(137) $$H=\int \left[\nu \right]d\Phi =\int_{\epsilon }^{a}d\bar{r}\int_{0}^{2\pi }d\eta \Gamma_{L}\left(-\frac{1}{2\pi }\partial_{\bar{r}}\Gamma_{S}\left(\bar{r}\right)\right)=\Gamma_{L}\left(\Gamma_{S}\left(\epsilon \right)-\Gamma_{S}\left(a\right)\right).$$

Thus the helicity is due to the azimuthal vortex lines surrounding a “virtual” vortex line along the symmetry axis of the torus (the author would like to thank Professor Moffatt for the interpretation of this result). Of course the helicity can also be derived using the standard Equation (74) but with an identical result.

### 12.3. Dynamics on the Torus

At this point we have only studied the flow kinematics on the said torus; we have not discussed the forces that cause the flow. The dynamics of the flow are encapsulated in a single scalar equation, which is the α Equation (63). Thus, the force potential needed to drive the flow is:

(138) $$\begin{array}{ccc} \;\Psi & = & \frac{1}{2}{\vec{v}}^{2}+w\left(\rho \right)-\alpha \\ \; & = & \frac{1}{2}\left({\left(\frac{\Gamma_{S}\left(\bar{r}\right)}{2\pi \bar{r}}\right)}^{2}+{\left(\frac{\Gamma_{L}}{2\pi R}\right)}^{2}\right)+w\left(\frac{g(\bar{r})}{R}\right)-\int_{\epsilon }^{\bar{r}}d{\bar{r}}^{\prime }\frac{\Gamma_{S}\left({\bar{r}}^{\prime }\right)\partial_{{\bar{r}}^{\prime }}\Gamma_{S}\left({\bar{r}}^{\prime }\right)}{{\left(2\pi {\bar{r}}^{\prime }\right)}^{2}}. \end{array}$$

for which we have used Equations (118), (121) and (131) and also taken into account that the specific entropy *s* is uniform. It follows that the force potential needed to support this family of flows depends on the equation of state w(ρ,s) of the material under consideration, in addition to the arbitrary functions $\Gamma_{S}\left(\bar{r}\right),g\left(\bar{r}\right)$ and the constant $\Gamma_{L}$. It is obvious that the validity of this family of solutions can be verified by inserting the velocity field given in Equation (121) and the density of Equation (118) into Euler Equation (2) and the continuity Equation (1).

We consider the case in which Ψ is gravitational. Two possible cases may be considered: the case of torus self-gravity, in which the potential Ψ must satisfy:

(139) $${\vec{\nabla }}^{2}\Psi =4\pi G\rho =4\pi G\frac{g(\bar{r})}{R},$$

where *G* is the universal constant of gravity, and the case in which Ψ is external, such that the potential Ψ must satisfy:

(140) $${\vec{\nabla }}^{2}\Psi =0,$$

inside the torus. The potential is a function of $\bar{r},\eta$ made of two contributions:

(141) $$\begin{array}{ccc} \Psi (\bar{r},\eta ) & = & \Psi_{1}\left(\bar{r}\right)+\Psi_{2}\left(\bar{r},\eta \right),\\ \;\Psi_{1}\left(\bar{r}\right) & \equiv & \frac{1}{2}{\left(\frac{\Gamma_{S}\left(\bar{r}\right)}{2\pi \bar{r}}\right)}^{2}-\int_{\epsilon }^{\bar{r}}d{\bar{r}}^{\prime }\frac{\Gamma_{S}\left({\bar{r}}^{\prime }\right)\partial_{{\bar{r}}^{\prime }}\Gamma_{S}\left({\bar{r}}^{\prime }\right)}{{\left(2\pi {\bar{r}}^{\prime }\right)}^{2}},\\ \Psi_{2}\left(\bar{r},\eta \right) & \equiv & \frac{1}{2}{\left(\frac{\Gamma_{L}}{2\pi R}\right)}^{2}+w\left(\frac{g(\bar{r})}{R}\right). \end{array}$$

$\Psi_{2}\left(\bar{r},\eta \right)$ is required to balance pressure and centrifugal forces due to rotation the long way round the torus. Let us consider the case in which $\Psi_{1}\left(\bar{r}\right)>>\Psi_{2}\left(\bar{r},\eta \right)$ (pressure and “long-way” centrifugal forces neglected) such that:

(142) $$\Psi ≃\Psi_{1}\left(\bar{r}\right).$$

Using Equation (102), Equation (139) takes the approximated form:

(143) $$\partial_{\bar{r}}^{2}\Psi +\frac{2}{\bar{r}}\partial_{\bar{r}}\Psi -\frac{1}{R}\left(\frac{1}{\bar{r}}\partial_{\bar{r}}\Psi \right)=4\pi G\frac{g(\bar{r})}{R}.$$

A solution is possible only if the following is satisfied:

(144) $$\partial_{\bar{r}}^{2}\Psi +\frac{2}{\bar{r}}\partial_{\bar{r}}\Psi =0.$$

This is solved by:

(145) $$\Psi =\frac{C_{1}}{\bar{r}}+C_{2}.$$

in which $C_{1},C_{2}$ are constants. Hence:

(146) $$g\left(\bar{r}\right)=\frac{C_{1}}{4\pi G{\bar{r}}^{3}}.$$

Through Equation (141) we can derive the circulation round the short way:

(147) $$\Gamma_{S}\left(\bar{r}\right)=2\pi \sqrt{C_{1}\bar{r}}.$$

Possible applications of tori in physics are well known. Tokamaks used for fusion research are basically plasma tori. However, they are not self-gravitating tori. The rings of Saturn are well-known astrophysical objects. However, they are also not self-gravitating because they are highly affected by the gravitational field of the planet Saturn. Self-gravitating astrophysical disks are used to model galaxies, and non-self-gravitating disks are also studied, for example in the case of accretion disks, but disks have a fundamentally different topology than tori. Hence, self-gravitating tori are novel astrophysical objects and their existence (or lack of existence) needs to be confirmed by astronomers.

### Summary

We show how to the variational variables are useful for obtaining analytical solutions of the flow equations; in particular we derive a solution describing a self-gravitating torus.

## 13. Conclusions

We have discussed the variational analysis of non-barotropic flows, starting from a classical Lagrangian variational analysis and moving later to an Eulerian variational analysis, which demanded auxiliary functions. In the Eulerian case the Lagrangian depends on nine functions: the original set of five functions $\rho ,\vec{v},s$ and auxiliary variables α,β,ν,σ. This was later reduced to a six-function Lagrangian depending on α,β,ν,σ,ρ,s. The Eulerian variational description was further reduced to a four-function Lagrangian with the remaining variables β,ν,ρ,s, leading to a set of four cumbersome equations. We have also dedicated a paragraph to the stationary version of our Lagrangian formalism. Our result can be compared with respect to what was achieved in the less realistic barotropic case; there a three function formalism is available for both stationary and non-stationary flows. In both barotropic and non-barotropic cases, the variational formalism has reduced the number of variables needed with respect to the physical description. The same equations can be obtained using either a Lagrangian formalism or Hamiltonian formalism provided that we adhere to a Eulerian description of the flow. However, this economy of variables comes with a topological cost. In the barotropic case the vorticity lines must lie on surfaces and cannot be volume-filling. This is also true for non-barotropic flows, in which one demands the same for the topological vorticity.

We would like to mention an excellent review paper that deals with conservation laws for CGL and MHD plasmas [20]. This paper discusses the cross-helicity conservation laws for a baroclinic gas with equation of state p=p(ρ,s). See also [21,22,23].

Analyzing the stability and describing numerical schemes using the discussed variational principles is beyond the scope of this paper. The current work is related to stability issues in two aspects: One of them is by showing that the dynamical space (the number of independent degrees of freedoms) is four-dimensional and not five, which inherently reduces the possible instabilities that can develop (see Section 11). The second has to do with conserved quantities, especially local conserved quantities like topological helicity per unit of topological vorticity flux given in Equation (89), which also inhibit instabilities from developing. It is likely that to address these aspects, we will need to introduce additional constants of motion constraints into the action, similarly to what was performed by Arnold and others [24,25] and also discussed in other works [26,27]. Additional points for future study include the Noether currents of the present variational formalism and their implications. Also it is possible that, as in the magnetohydrodynamic case, there may be a way to reduce the variable number down to three for non-barotropic stationary fluid dynamics. Hopefully this will be studied in a future paper.

## Data Availability

Data is contained within the article.

## Appendix A. Variational Formalism of Point Particles

Let us examine a classical particle with coordinates $\vec{x}\left(t\right)$ and mass *m* moving under the influence of an external force, which can be derived from a scalar potential $\Psi (\vec{x},t)$. We are not concerned with the particle’s influence on the field, treating the field as “external.” The action for this particle is given by:

(A1) $$\begin{array}{ccc} A & = & \int_{t1}^{t2}Ldt,\\ L & \equiv & \frac{1}{2}mv^{2}-m\Psi ,\;\vec{v}\equiv \frac{d\vec{x}}{dt}\equiv \dot{\vec{x}},\;v=\left|\vec{v}\right|. \end{array}$$

Varying the Lagrangian:

(A2) $$\delta L=m\dot{\vec{x}}\cdot \delta \dot{\vec{x}}-m\vec{\nabla }\Psi \cdot \delta \vec{x}=\frac{d(m\vec{v}\cdot \delta \vec{x})}{dt}-m\dot{\vec{v}}\cdot \delta \vec{x}-m\vec{\nabla }\Psi \cdot \delta \vec{x}.$$

Thus:

(A3) $$\delta L=\frac{d\left(m\vec{v}\cdot \delta \vec{x}\right)}{dt}+\left(-m\dot{\vec{v}}+\vec{F}\right)\cdot \delta \vec{x}.\;\vec{F}\equiv -m\vec{\nabla }\Psi .$$

This entails a variation in the action:

(A4) $$\delta A=\int_{t1}^{t2}\delta Ldt={\left.m\vec{v}\cdot \delta \vec{x}\right|}_{t1}^{t2}-\int_{t1}^{t2}\left(m\dot{\vec{v}}-\vec{F}\right)\cdot \delta \vec{x}dt.$$

Since the classical trajectory is defined by the principle that any small variation in the action $\delta \vec{x}$ (which vanishes at times $t_{1}$ and $t_{2}$ but is otherwise arbitrary) results in no change in the action, it follows that:

(A5) $$m\dot{\vec{v}}=\vec{F}.$$

For a system consisting of *N* particles, where each particle is identified by an index 1≤j≤N, with corresponding mass $m_{j}$, charge $e_{j}$, position vector ${\vec{x}}_{j}$, and velocity ${\vec{v}}_{j}\equiv \frac{d{\vec{x}}_{j}}{dt}$, the action and Lagrangian for each particle are given as follows:

(A6) $$\begin{array}{ccc} A_{j} & = & \int_{t1}^{t2}L_{j}dt,\\ L_{j} & \equiv & \frac{1}{2}m_{j}v_{j}^{2}-m_{j}\Psi_{j}. \end{array}$$

From the above it is easy to derive the action and Lagrangian for a system of particles as:

(A7) $$A_{s}=\int_{t1}^{t2}L_{s}dt,\;L_{s}=\sum_{j=1}^{N}L_{j}.$$

The variational analysis follows along the same lines as for a single particle. We thus obtain the following set of equations:

(A8) $$m_{j}{\dot{\vec{v}}}_{j}={\vec{F}}_{j},\;j\in \left[1-N\right].$$

## Appendix B. Variation in the Volume Element

Let us calculate the variation in the volume element, taking into account Equation (27):

(A9) $$\begin{array}{ccc} \Delta dV_{\vec{\alpha },t} & = & \Delta d^{3}x\left(\vec{\alpha },t\right)=\Delta J\;d^{3}x\left(\vec{\alpha },0\right)=\frac{\Delta J}{J}d^{3}x\left(\vec{\alpha },t\right)=\frac{\Delta J}{J}dV_{\vec{\alpha },t},\\ (\Delta d^{3}x\left(\vec{\alpha },0\right) & = & 0). \end{array}$$

Here both the actual and varied trajectories start at the same location. The reader is referred to [28] for a precise definition of Eulerian and Lagrangian variations. Varying *J* we obtain

(A10) $$\Delta J={\vec{\nabla }}_{0}\Delta x_{1}\cdot \left({\vec{\nabla }}_{0}x_{2}\times {\vec{\nabla }}_{0}x_{3}\right)+{\vec{\nabla }}_{0}x_{1}\cdot \left({\vec{\nabla }}_{0}\Delta x_{2}\times {\vec{\nabla }}_{0}x_{3}\right)+{\vec{\nabla }}_{0}x_{1}\cdot \left({\vec{\nabla }}_{0}x_{2}\times {\vec{\nabla }}_{0}\Delta x_{3}\right).$$

Introducing the notation $\vec{\xi }\equiv \Delta \vec{x}$ it follows that:

(A11) $$\begin{array}{ccc} & & {\vec{\nabla }}_{0}\Delta x_{1}\cdot \left({\vec{\nabla }}_{0}x_{2}\times {\vec{\nabla }}_{0}x_{3}\right)={\vec{\nabla }}_{0}\xi_{1}\cdot \left({\vec{\nabla }}_{0}x_{2}\times {\vec{\nabla }}_{0}x_{3}\right)\\ & = & \partial_{k}\xi_{1}{\vec{\nabla }}_{0}x_{k}\cdot \left({\vec{\nabla }}_{0}x_{2}\times {\vec{\nabla }}_{0}x_{3}\right)=\partial_{1}\xi_{1}{\vec{\nabla }}_{0}x_{1}\cdot \left({\vec{\nabla }}_{0}x_{2}\times {\vec{\nabla }}_{0}x_{3}\right)=\partial_{1}\xi_{1}J.\\ & & {\vec{\nabla }}_{0}x_{1}\cdot \left({\vec{\nabla }}_{0}\Delta x_{2}\times {\vec{\nabla }}_{0}x_{3}\right)={\vec{\nabla }}_{0}x_{1}\cdot \left({\vec{\nabla }}_{0}\xi_{2}\times {\vec{\nabla }}_{0}x_{3}\right)\\ & = & \partial_{k}\xi_{2}{\vec{\nabla }}_{0}x_{1}\cdot \left({\vec{\nabla }}_{0}x_{k}\times {\vec{\nabla }}_{0}x_{3}\right)=\partial_{2}\xi_{2}{\vec{\nabla }}_{0}x_{1}\cdot \left({\vec{\nabla }}_{0}x_{2}\times {\vec{\nabla }}_{0}x_{3}\right)=\partial_{2}\xi_{2}J.\\ & & {\vec{\nabla }}_{0}x_{1}\cdot \left({\vec{\nabla }}_{0}x_{2}\times {\vec{\nabla }}_{0}\Delta x_{3}\right)={\vec{\nabla }}_{0}x_{1}\cdot \left({\vec{\nabla }}_{0}x_{2}\times {\vec{\nabla }}_{0}\xi_{3}\right)\\ & = & \partial_{k}\xi_{3}{\vec{\nabla }}_{0}x_{1}\cdot \left({\vec{\nabla }}_{0}x_{2}\times {\vec{\nabla }}_{0}x_{k}\right)=\partial_{3}\xi_{3}{\vec{\nabla }}_{0}x_{1}\cdot \left({\vec{\nabla }}_{0}x_{2}\times {\vec{\nabla }}_{0}x_{3}\right)=\partial_{3}\xi_{3}J. \end{array}$$

Thus:

(A12) $$\Delta J=\partial_{1}\xi_{1}J+\partial_{2}\xi_{2}J+\partial_{3}\xi_{3}J=\vec{\nabla }\cdot \vec{\xi }\;J.$$
